# Trends of Enteric Fever and Emergence of Extensively Drug-Resistant Typhoid in Pakistan: Population-Based Laboratory Data From 2017–2019

**DOI:** 10.1093/ofid/ofaf106

**Published:** 2025-03-05

**Authors:** Farah Naz Qamar, Mohammad Tahir Yousafzai, Ibtisam Qazi, Sonia Qureshi, Naor Bar-Zeev, Shazia Sultana, Muhammad Jawwad, Aneeta Hotwani, Seema Irfan, Muhammad Ashraf Memon, Irim Iftikhar, Summiya Nizamuddin, Ikram Ujjan, Ejaz Ahmed Khan, Mohsina Noor Ibrahim

**Affiliations:** Department of Paediatrics & Child Health, Aga Khan University, Karachi, Pakistan; Department of Paediatrics & Child Health, Aga Khan University, Karachi, Pakistan; School of Population Health, UNSW Sydney, Sydney, Australia; Department of Paediatrics & Child Health, Aga Khan University, Karachi, Pakistan; Department of Paediatrics & Child Health, Aga Khan University, Karachi, Pakistan; International Vaccine Access Center, Department of International Health, John Hopkins Bloomberg School of Public Health, Baltimore, Maryland, USA; Department of Paediatrics & Child Health, Aga Khan University, Karachi, Pakistan; Department of Paediatrics & Child Health, Aga Khan University, Karachi, Pakistan; Department of Paediatrics & Child Health, Aga Khan University, Karachi, Pakistan; Department of Pathology and Laboratory Medicine, The Aga Khan University, Karachi, Pakistan; Kharadar General Hospital, Karachi, Pakistan; Chughtai Laboratory, Lahore, Pakistan; Section of Microbiology, Department of Pathology, Shaukat Khanum Memorial Cancer Hospital and Research Centre, Lahore, Pakistan; LUHMS Laboratory, Hyderabad, Pakistan; Shifa International Hospital, Shifa Tameer-e-Millat University, Islamabad, Pakistan; National Institute of Child Health, Karachi, Pakistan

**Keywords:** antibiotics, blood cultures, extensively drug resistant, multidrug resistant, antimicrobial resistance, Salmonella Typhi and Paratyphi

## Abstract

**Background:**

Typhoid fever burdens low- and middle-income countries, especially children. Despite being curable, it now resists first-line antibiotics. This study aims to understand antimicrobial resistance patterns associated with multidrug-resistant (MDR) and extensively drug-resistant (XDR) typhoid fever cases in Pakistan.

**Methods:**

We conducted a retrospective review of blood culture–confirmed typhoid cases from 5 large laboratory networks in Pakistan over a period of 3 years (2017–2019). Data were analyzed for 464 956 blood culture specimens, of which Typhi and Paratyphi were isolated in 23 924 (5%) of all blood cultures done.

**Results:**

Sindh had the highest proportion of *S.* Typhi cases (72%) of all positive cases, followed by Punjab with 46.9%. The 5–14-years age group had the highest proportion of *S.* Typhi (MDR: 46.1%; XDR: 44.2%), followed by the 2–4-years age group (MDR: 27%; XDR: 26.2%). XDR isolates of *S.* Typhi were found in 57%. Most *S.* Typhi isolates were resistant to ampicillin (79.8%), chloramphenicol (80.8%), cefixime (64.6%), ciprofloxacin (66.4%), ceftriaxone (63.3%), and co-trimoxazole (80.2%). Most *S.* Paratyphi isolates were responsive to antibiotics, ampicillin (97.2%), chloramphenicol (98.6%), cefixime (99.5%), ceftriaxone (99.5%), and co-trimoxazole (98.7%). Resistance to ciprofloxacin was 85.9%. Both *S.* Typhi and *S.* Paratyphi were susceptible to azithromycin and imipenem, whereas 99.8% of *S.* Typhi and 100% of *S.* Paratyphi were sensitive to meropenem.

**Conclusions:**

Increased prevalence of culture-confirmed XDR *S.* Typhi cases was observed in 2019 as compared with 2017, presumably due to the outbreak of XDR in Sindh.

## Teaser

A large-scale review of blood culture-confirmed typhoid cases in Pakistan (2017–2019) revealed a high prevalence of extensively drug-resistant (XDR) Salmonella Typhi, particularly in Sindh, where 57% of isolates were XDR. Alarmingly, most S. Typhi isolates showed resistance to multiple first-line antibiotics, while S. Paratyphi remained largely susceptible. These findings highlight the urgent need for targeted interventions to curb the spread of drug-resistant typhoid.

Enteric fever is caused by the gram-negative bacterium *Salmonella enterica* serovar Typhi and Paratyphi A and B. Globally, there has been a decline in the burden of typhoid and paratyphoid fever from 25.9 million cases in 1990 to 14.3 million cases in 2017 [1]. This decline is mostly due to elimination from high-income countries. The disease is still prevalent in regions across South Asia, Southeast Asia, and Africa, primarily attributed to inadequate WASH (water, sanitation, and hygiene) conditions. The high burden in these regions, coupled with a rise in antimicrobial resistance, presents a challenge for determining disease burden and treatment.

During the Surveillance for Enteric Fever in Asia Project (SEAP) conducted from 2016 to 2019, the reported adjusted *S.* Typhi incidences in public sector facilities in Pakistan were 176 and 103 (per 100 000 person-years) and 23 and 1 (per 100 000 person-years), respectively [2]. Extensively drug-resistant (XDR) typhoid was 64% among *S.* Typhi cases in Pakistan during the SEAP study period [3, 4].

While SEAP provides burden estimates for Sindh, credible and comparable estimates for the rest of the country, including the densely populated province of Punjab, are currently unavailable. Existing data primarily originate from single centers situated in various regions, highlighting a substantial burden of typhoid fever. However, the applicability of data derived from individual centers may be limited in terms of usefulness for the strategic planning of prevention programs. Establishing and maintaining a robust surveillance system in countries like Pakistan pose considerable challenges. An alternative approach involves leveraging data from extensive laboratory networks equipped with satellite facilities across the country. Such data not only present a cost-effective method for assessing the burden of typhoid fever but also hold potential value in comprehending the evolving epidemiology of infectious diseases including *S.* Typhi and Paratyphi.

Therefore, this retrospective review of laboratory-based data was conducted to determine the epidemiology and antimicrobial resistance (AMR) of *S.* Typhi cases in Pakistan. The aim of this study was to report the prevalence and antimicrobial resistance patterns of strains of *S.* Typhi and *S.* Paratyphi cases using data from large public and private laboratory networks across Pakistan from 2017 to 2019.

## METHODS

### Study Design and Site Selection

We conducted a retrospective review of blood culture–confirmed *S.* Typhi cases from 5 large laboratory networks in Pakistan over a period of 3 years (2017–2019). Aga Khan University Hospital laboratory (AKUH) (a) is a private laboratory located in the metropolitan city of Karachi with >337 collection points all over the country. Liaquat University of Medical and Health Sciences (LUMHS) (b) is a public sector laboratory in Hyderabad city with 30 collection points in all major cities of Sindh. Chughtai laboratory (CLL) (c), situated in Lahore, has 328 satellite collection points in >100 cities across Pakistan. Shaukat Khanum Memorial Hospital and Research Center (SKMCH) (d), situated in Lahore, has a private laboratory with 174 collection points all over the country, and Shifa International Hospital (SIH) (e) is a private laboratory situated in Islamabad with some collection points in Punjab and Sindh. Chughtai and SKMCH laboratory networks have more collection points in Punjab province, AKUH has more in Sindh as compared with the rest of the country, and LUMHS has them only in Sindh. These laboratory networks were selected because of their wide network of satellite labs throughout the country, existence of quality assurance systems for validation of laboratory procedures, and availability of at least 3 years of retrospective records. These laboratories are also part of an ongoing prospective typhoid surveillance study of the impact of typhoid conjugate vaccine (TCV). Antibiotic susceptibility of the isolates was reported in accordance with Clinical laboratory Standards Institute (CLSI) guidelines for *Salmonella* isolates [6]. All laboratories used the Bactec platform for identification of blood cultures, except AKUH, which uses the Vitek platform (Supplementary Appendix 1, list of laboratories).

The exact catchment population of these 5 large-scale laboratory networks is dependent on the health care utilization of these labs in each part of the country (total Pakistan population as of 2017/2018 census = 207 684 626); however, in Sindh and Punjab provinces, these laboratory networks covers almost 90% of the population (Sindh = 47 854 510 and Punjab = 109 989 655).

### Data Extraction

From January 2017 to December 2019, all reports of blood cultures positive for *S.* Typhi or *S.* Paratyphi were extracted from the databases of the 5 laboratories. The following data were extracted for every case: specimen number, date of collection, location of the satellite lab (as a proxy for the case residence; total counts of blood cultures conducted monthly were all included), demographic data including age and gender, and antimicrobial sensitivity information. Data for antimicrobial resistance patterns were extracted for ampicillin, chloramphenicol, co-trimoxazole, ciprofloxacin, cefixime, ceftriaxone, azithromycin, and imipenem. *Salmonella* Typhi or Paratyphi A or B strains resistant or intermediate to ampicillin, chloramphenicol, and trimethoprim-sulphamethoxazole were classified as multidrug-resistant (MDR) cases. Isolates resistant to ampicillin, chloramphenicol, trimethoprim-sulphamethoxazole, fluoroquinolones, and ceftriaxone were termed XDR cases [5].

### Statistical Analysis

The data were cleaned and imported into STATA, version 14, for the analysis. Descriptive statistics were computed for both continuous and categorical variables. For continuous variables, means with SDs were calculated. Frequencies and percentages were calculated for categorical variables, such as sociodemographic characteristics, total number of blood cultures, number of blood cultures positive for *S.* Typhi and Paratyphi, MDR and XDR Typhi cases, and antimicrobial susceptibility. Data were stratified to show changes in the trends of incident cases monthly for Sindh, Punjab, and the other 2 provinces combined (Baluchistan and Khyber Pakhtunkhwa). The number of blood cultures that were positive for *S.* Typhi and Paratyphi was estimated collectively for all laboratories and for individual laboratories by taking out the number of blood culture–confirmed cases from the total number of blood cultures performed per month. The proportions of *S.* Typhi and *S.* Paratyphi cases among all positive cases and those of MDR, XDR, and fluoroquinolone resistant (FQR) cases were also computed.

### Patient Consent

The study protocol was reviewed and approved by the ethical review committee of Aga Khan University, Karachi, and the National Bioethics Committee of Pakistan. Ethical approvals were also obtained from the ethical review committees of LUMHS, CLL, SKMCH, and Shifa laboratories. Informed consent was exempted due to the retrospective nature of the data. All data shared by the laboratories were deidentified. Data were stored in password-protected computers on a secured central server system and were accessible only to senior members of the study team.

## RESULTS

Data for a total of 464 956 blood culture were available from 5 major laboratories across Pakistan for the period between January 2017 and December 2019. Among all the blood cultures conducted, confirmed cases of enteric fever were 23 924 (5%). Most positive blood cultures for *S.* Typhi and *S.* Paratyphi were from AKUH, accounting for 13 979 cases (58.4%), followed by LUMHS with 4104 cases (17.2%), CCL with 2736 cases (11.4%), SKMCH with 2186 cases (9.1%), and SIH with 919 cases (3.8%). Within this cohort, the predominant cases were of *S.* Typhi, comprising 21 864 cases (91.4%) of the total burden, followed by *S.* Paratyphi A at 2040 cases (8.5%), with only 20 cases (0.1%) attributed to *S.* Paratyphi B. An increasing trend in the number of culture-confirmed *S.* Typhi cases was observed in 2019 as compared with 2017, with XDR isolates contributing to most of these cases. In contrast, the proportions of *S.* Paratyphi remained the same over the 3 years (Table 1).

**Table 1.** Blood Culture–Confirmed Cases of *S.* Typhi and Paratyphi From Five Laboratory Networks Across Pakistan (2017–2019)

Year	Total Blood Cultures Performed	Proportion of Cultures Positive for *S.* Typhi or Paratyphi, No. (%)	*S.* Typhi, No. (%)	*S.* Paratyphi, No. (%)	MDR (*S.* Typhi), No. (%)	XDR (*S.* Typhi), No. (%)
2017	112 244	3143 (2.8)	2543 (2.3)	600 (0.5)	31 (1.2)	718 (28.2)
2018	132 948	5567 (4.2)	5215 (3.9)	352 (0.3)	56 (1.1)	3272 (62.7)
2019	219 764	15 214 (6.9)	14 106 (6.4)	1108 (0.5)	143 (1.0)	9026 (64)

Abbreviations: MDR, multidrug resistant; XDR, extensively drug resistant.

**Figure 1.**
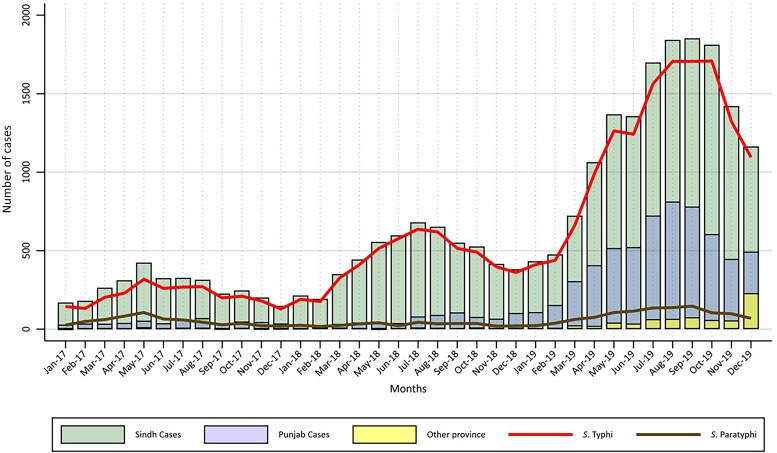
Monthly trends of blood culture enteric fever cases by province from 2017–2019.

A seasonal trend was observed, with the highest case numbers being reported from April to October for all 3 years in Sindh and for 2019 in Punjab. An overall increase in blood culture–confirmed *S.* Typhi cases was recorded in Sindh over 3 years, with a steep rise in cases witnessed in 2019 as compared with the previous 2 years (Figure 1).

Table 2 describes the sociodemographic characteristics of culture-confirmed enteric fever cases. Among all cultures confirmed for typhoid, children aged 5–14 years accounted for the highest proportion (43.6), followed by those aged 2–4 years (22.9%). Stratified provincial data revealed that the highest proportion of *S.* Typhi cases (72%) was reported from Sindh. Punjab had a higher number of *S.* Paratyphi cases (46.9%) compared with *S.* Typhi cases.

**Table 2.** Sociodemographic Characteristics of Blood Culture–Confirmed Enteric Fever Cases From Five Laboratory Networks Across Pakistan (2017–2019)

	Total	*S.* Typhi^a^	*S.* Paratyphi	MDR (*S.* Typhi)	XDR (*S.* Typhi)
	23 924	21 864	2060	230	13 016
	No. (%)	No. (%)	No. (%)	No. (%)	No. (%)
Age					
<2 y	1954 (8.2)	1916 (8.8)	38 (1.8)	22 (9.6)	1341 (10.3)
2–4 y	5484 (22.9)	5284 (24.2)	200 (9.7)	62 (27.0)	3411 (26.2)
5–14 y	10 427 (43.6)	9745 (44.6)	682 (33.1)	106 (46.1)	5747 (44.2)
15–24 y	3465 (14.5)	2953 (13.5)	512 (24.9)	34 (14.8)	1589 (12.2)
≥25 y	2594 (10.8)	1966 (9.0)	628 (30.5)	6 (2.6)	928 (7.1)
Gender					
Males	14 359 (60.0)	13 065 (59.8)	1294 (62.8)	119 (51.7)	7895 (60.7)
Females	9565 (40.0)	8799 (40.2)	766 (37.2)	111 (48.3)	5121 (39.3)
Province					
Sindh	16 784 (70.2)	15 743 (72.0)	1041 (50.5)	217 (94.3)	10 271 (78.9)
Punjab	6286 (26.3)	5319 (24.3)	967 (46.9)	12 (5.2)	2274 (17.5)
Other provinces	854 (3.6)	802 (3.7)	52 (2.5)	1 (0.4)	471 (3.6)

Abbreviations: FQR, fluoroquinolone resistant; MDR, multidrug resistant; XDR, extensively drug resistant.

^a^The rest of the case numbers comprised MDR-Cipro, FQR, sensitive, any resistance other than MDR or XDR, and missing data on antibiotic sensitivity.

**Figure 2.**
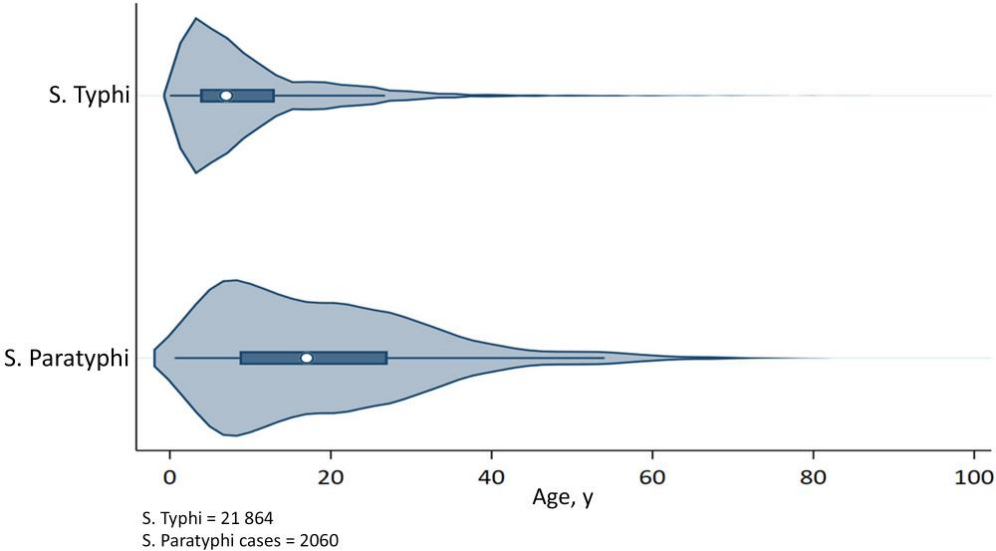
Age distribution of S. Typhi and Paratyphi cases.

**Figure 3.**
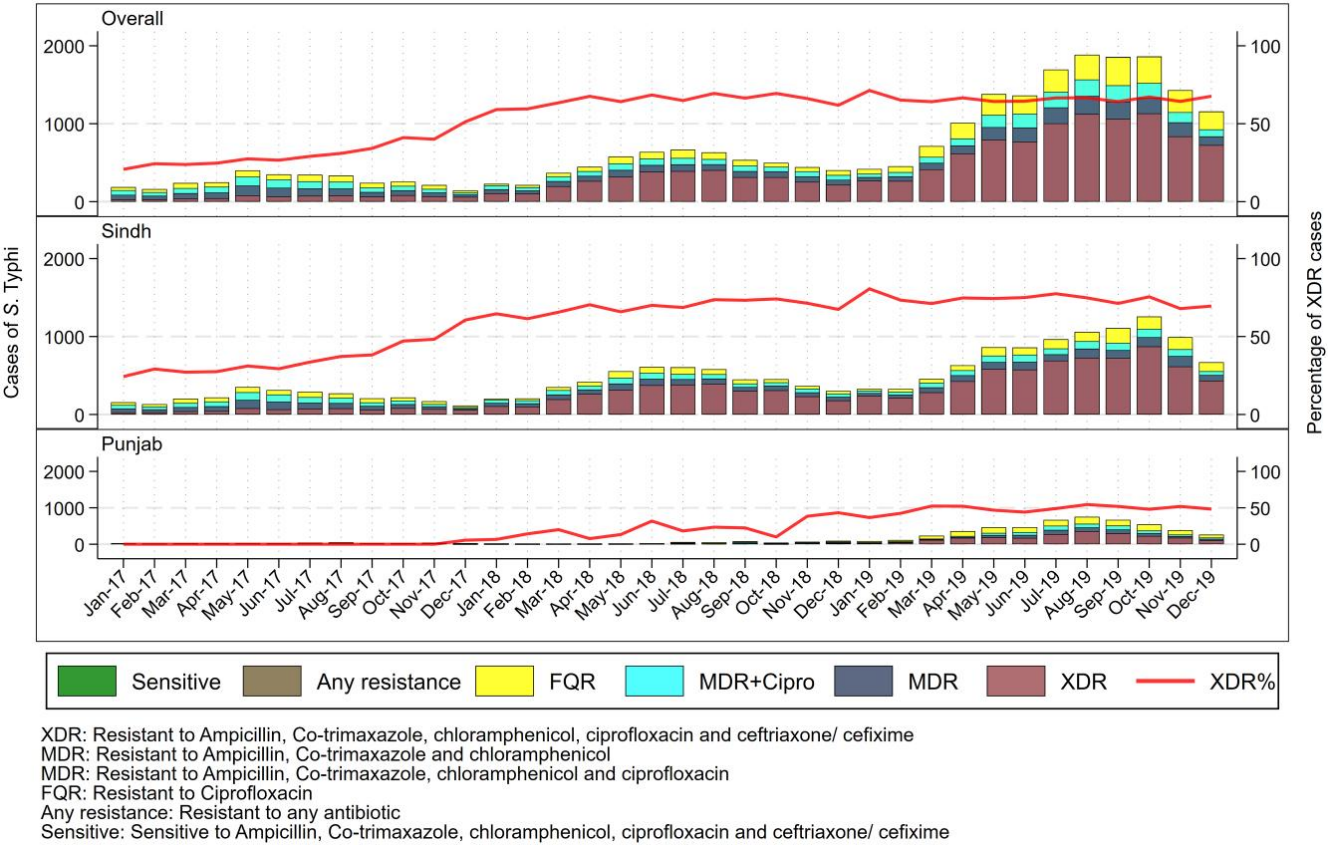
Monthly trends in AMR resistance reported in S. Typhi cases from 2017–2019. XDR: resistant to ampicillin, co-trimaxazole, chloramphenicol, ciprofloxacin, and ceftriaxone/cefixime. MDR: resistant to ampicillin, co-trimaxazole, and chloramphenicol. MDR+Cipro: resistant to ampicillin, co-trimaxazole, chloramphenicol, and ciprofloxacin. FQR: resistant to ciprofloxacin. Any resistance: resistant to any antibiotic. Sensitive: sensitive to ampicillin, co-trimaxazole, chloramphenicol, ciprofloxacin, and ceftriaxone/cefixime. Abbreviations: FQR, fluoroquinolone resistant; MDR, multidrug resistant; XDR, extensively drug resistant.

**Figure 4.**
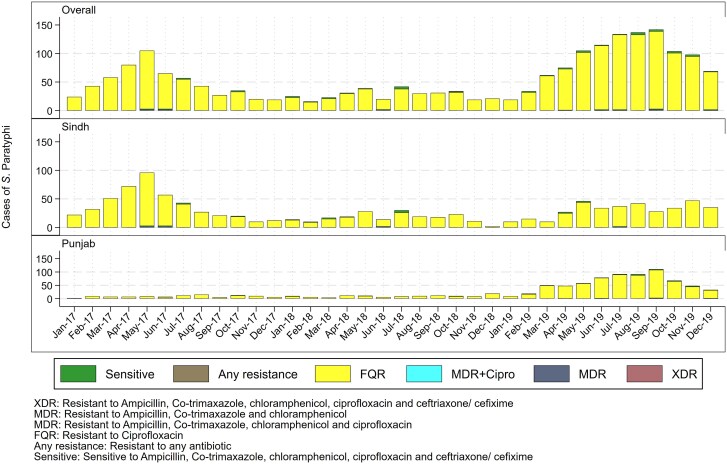
Monthly trends in AMR resistance reported in S. Typhi cases from 2017–2019. XDR: resistant to ampicillin, co-trimaxazole, chloramphenicol, ciprofloxacin, and ceftriaxone/cefixime. MDR: resistant to ampicillin, co-trimaxazole, and chloramphenicol. MDR+Cipro: resistant to ampicillin, co-trimaxazole, chloramphenicol, and ciprofloxacin. FQR: resistant to ciprofloxacin. Any resistance: resistant to any antibiotic. Sensitive: sensitive to ampicillin, co-trimaxazole, chloramphenicol, ciprofloxacin, and ceftriaxone/cefixime. Abbreviations: FQR, fluoroquinolone resistant; MDR, multidrug resistant; XDR, extensively drug resistant.

The mean age for all culture-confirmed cases (SD) was 11.4 (10.7) years, with males (60%) being more affected as compared with females. Age densities with respect to organisms are depicted in Figure 2. *S.* Typhi was found to be more common in children younger than 15 years of age, while *S.* Paratyphi was found across a range of age groups, with a higher number of cases in those aged >14 years. Trends in AMR among cultures confirmed that *S.* Typhi showed increasing AMR, with the number of XDR *S.* Typhi cases increasing exponentially in 2019 (Figure 3). Among *S.* Paratyphi cases, an increasing number of cases resistant only to fluoroquinolone were also detected during the same period (Figure 4).

Out of all the cultures done, 57% were extensively drug-resistant isolates. The majority of the *S.* Typhi isolates were resistant to ampicillin (79.8%), chloramphenicol (80.8%), cefixime (64.6%), ciprofloxacin (66.4%), ceftriaxone (63.3%), and co-trimoxazole (80.2%) (Figure 3). Most *S.* Paratyphi isolates were sensitive to first-line antibiotics like ampicillin (97.2%), chloramphenicol (98.6%), cefixime (99.5%), ceftriaxone (99.5%), and co-trimoxazole (98.7%) and intermediately resistant to ciprofloxacin (85.9%) (Figure 4). All isolates of both *S.* Typhi and Paratyphi were susceptible to azithromycin (100%), imipenem (100%), and meropenem (*S.* Typhi: 99.8%; *S.* Paratyphi: 100%) (Figures 3 and 4).

## DISCUSSION

Our study reveals a concerning rise in the proportion of XDR typhoid cases. Resistance to first-line antibiotics and fluoroquinolones such as ciprofloxacin was found in a majority of *S.* Typhi isolates. The increasing trend in XDR and MDR cases over the past 3 years in Pakistan is alarming, and changing AMR patterns may have significant implications for available treatment options as drug resistance may render this largely curable and nonfatal condition untreatable. Intercontinental transmission of XDR typhoid linked to the outbreak in Sindh has also been reported from several countries such as Australia, Canada, Denmark, Taiwan, the United Kingdom, and the United States [7, 8]. The potential of geographic spread and the diminishing array of treatment options underscore the need for ongoing surveillance to comprehend the evolving dynamics of AMR in countries where typhoid is endemic. This has significant implications for both devising and implementing large-scale strategies for prevention. The swiftly changing dynamics of XDR transmission will substantially influence the cost of treatment and the severity of the disease [9].

Our retrospective review of laboratory-based data has attempted to elucidate these changing trends by providing critical information on AMR susceptibility patterns for culture-confirmed *S.* Typhi and Paratyphi cases from Pakistan. Even though *S.* Paratyphi has shown no resistance to first-line antibiotics except ciprofloxacin, it is still essential to monitor trends in AMR susceptibility patterns to avoid the future emergence of drug resistance to this strain. Similarly, unlike Typhi, the proportion of Paratyphi is higher in older age (88.5% Paratyphi in the 5–25-and-above age group vs 67.1% Paratyphi in the same age group) as compared with Typhi, which is higher in younger age (68.8% Typhi in the up-to-14-years age group vs 44.6% Paratyphi in the same age group). This has implications for the vaccination program. The optimal age range for the introduction of vaccine against Typhi and Paratyphi (though Paratyphi vaccine is not available yet) in routine immunization needs to be customized accordingly. While the introduction of typhoid vaccination, for example, TCV in routine immunization, is effective among children younger than 5 years of age, Paratyphi vaccine, on the other hand, should be prioritized for children beyond the age of 5. This highlights the significance of age-appropriate vaccination procedures in controlling enteric fever.

Typhoid conjugate vaccine, prequalified by the World Health Organization, is a monovalent vaccine that only confers immunity against *S.* Typhi. In 1 review, the frequency of typhoid fever varied greatly between age groups. About 35% of the cases that were found involved children between the ages of 2 and 5 years, while the remaining 78% involved children between the ages of 2 and 10 years. Data from multiple countries reflect that the peak age of typhoid incidence is often 5–9 years, rather than a consistent trend of higher burden in younger children [10].

A disproportionately high burden of XDR typhoid was reported from 2 provinces in Pakistan, that is, Sindh and Punjab, especially in children. A previous study from Punjab reported similar findings, with XDR typhoid cases being 0% in 2017 and gradually increasing to 30% in 2018 and 50% in 2019, with the burden being highest in children [11]. The findings from our study are also consistent with studies from Bangladesh, India, and Vietnam, where high incidences of typhoid fever were found in children <15 years of age [12–14]. Despite these findings, the increased case numbers from our study can be explained by the typhoid outbreak that occurred in 2017, improved surveillance, and awareness of blood culture testing resulting in greater numbers of culture-confirmed cases being detected [15].

A distinct seasonal variation was also observed, with the highest number of cases consistently being reported from April to October. The period from December to February showed a low caseload over the 3-year period. Similar studies from Pakistan have reported an increased incidence of typhoid fever in the summer months, especially July to September [16]. Robust surveillance systems targeted at seasonal variation and local epidemiology can be vital for early detection of outbreaks [4].

A major strength of the study is the availability of data related to culture-confirmed cases and AMR susceptibility patterns spanning a period of 3 years from 5 laboratory networks with collection points all over Pakistan. To our knowledge, this is the first study that has attempted to fill in the gaps in evidence that exist for culture-confirmed cases of typhoid fever and AMR susceptibility patterns on a large scale. The use of laboratory-based data has proven to be a cost-effective method for measuring the burden of disease. A limitation of our study is the availability of limited data from Baluchistan and Khyber Pakhtunkhwa. Therefore, robust surveillance expanded to areas with low coverage, coupled with clinical correlation, should be done to understand the different factors associated with the disease so that a policy targeted at prevention and treatment of resistant strains can be formulated. The inability to connect clinical severity of typhoid cases to vaccination status is another limitation of the study.

## CONCLUSIONS

The 3-year retrospective data from our study show an increasing trend in XDR *S.* Typhi cases with extensive resistance to first-line antibiotics. *S.* Paratyphi cases were a small proportion of the total cases and were sensitive to commonly used antibiotics for typhoid.

## Supplementary Material

ofaf106_Supplementary_Data

## Supplementary Data

Supplementary materials are available at *Open Forum Infectious Diseases* online. Consisting of data provided by the authors to benefit the reader, the posted materials are not copyedited and are the sole responsibility of the authors, so questions or comments should be addressed to the corresponding author.
