# Supplementary figures and images for: Trends of Enteric Fever and Emergence of Extensively Drug-Resistant Typhoid in Pakistan: Population-Based Laboratory Data From 2017–2019
Source: Open Forum Infect Dis. 2025 Mar 5;12(4):ofaf106. doi: 10.1093/ofid/ofaf106 (PMC11982669; doi:10.1093/ofid/ofaf106)

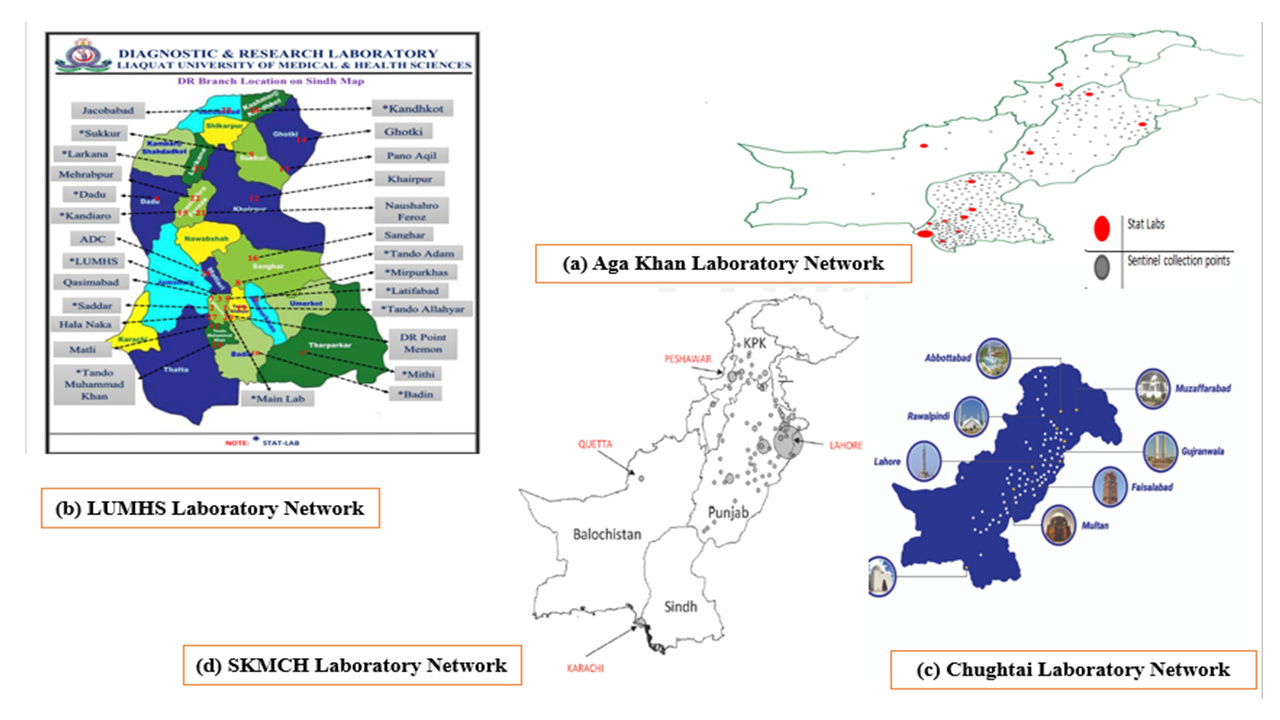

Supplement: ofaf106_Supplementary_Data [file ofaf106_supplementary_data.zip › appendix.tif]
